# The Mediator Role of Attitudes in Fish Choice Behavior: A Turkish Market Survey

**DOI:** 10.3390/foods11203180

**Published:** 2022-10-12

**Authors:** Osman Inanç Güney, Ilgın Özşahinoğlu, Zeynep Erçen, Hacer Yeldan, Çiğdem Dikel, Levent Sangün

**Affiliations:** 1 Vocational School of Adana, Cukurova Universitesi, Adana 01330, Turkey; 2 Marine Biology, Basic Fisheries Science, Cukurova University, Adana 01330, Turkey; 3 Department of Biotechnology, Cukurova University, Adana 01330, Turkey

**Keywords:** fish choice parameters, attitudes, socio-economic features, consumer-seller determinants

## Abstract

Due to the dynamic nature of demand, it is becoming increasingly important for the fish industry to investigate the changing choice behaviors of consumers in the face of increasing demand. This research investigated the role of attitudes and socio-demographic characteristics, which are the main factors in the fish choice behavior of consumers and in fish consumption behavior. In this context, an ordered probit model was constructed to analyze the effect of attitudes and socio-demographic characteristics as independent variables on fish consumption and purchase intention as the dependent variables. In addition, descriptive statistics were also used to reveal the current preferences related to fish. The data required for the model and descriptive statistics were obtained from 421 participants using a cross-sectional consumer survey covering the main cities of the seven regions of Turkey. The results show that while consumers prefer fish more than red meat and less than poultry, they mostly buy fresh fish from fish markets. Moreover, taste, physical appearance, convenience, wild fish, and seller trust attitudes have a significant and positive relationship with the dependent variable (the frequency of fish purchase and consumption) and price has a negative and significant relationship. Moreover, an increase in education level has a positive and significant relationship with the frequency of fish consumption. The research results provide important suggestions and information for decision-makers in the fish industry to implement effective policies and meet the consumer expectations of producers and distributors in the fish industry. In addition, the current study provides guidance for future research.

## 1. Introduction

### 1.1. The Food Choice Behavior Phenomenon

Food choice is a dynamic, multidimensional, and complex phenomenon influenced by many interconnected factors; so, it has been the focus of food market research analysis over the last 50 years [1,2,3,4,5]. The studies of [6], who calculated that consumer make more than 220 food-related decisions per day, demonstrate the great frequency and significance of this choice. The authors of [7] suggested that individuals’ food choices are influenced by three categories: (i) the nutritional content and physical properties of the food, (ii) sensory attributes (taste, odor, texture, etc.) and psychological factors of the individuals, and (iii) the economic and social environment. Factors that influence food choice can also be summarized as sensory factors (taste, odor, and texture) and non-sensory factors (price, health claims, availability, sustainability, convenience, personal values, etc.) [8].

Food choice behavior has also been affected by economic and social drivers such as increases in per capita income, changes in agri-food sector practices, diversification of production methods, fluctuating food prices, trade liberalization, supply chain developments, and the increased role of the media and marketing over the past several decades [9,10,11,12].

The importance of the influencing categories is likely to vary between consumers and products, making it crucial to understand which factors are important among specific segments for evaluating food choice and the drivers behind it [13].

Revealing the factors underlying consumers’ food preferences is one of the main issues that agri-food marketing experts have focused on for many years. Globalization and developments in communication opportunities have affected the speed and dynamics of changes in food preferences. Therefore, the scope of research in this area is expanding, and the frequency of updates is increasing.

### 1.2. Attitudes toward Fish Purchase and Consumption Intentions

In marketing, understanding attitudes can help marketers predict consumer behavior [14]. The differences in attitudes are reflected in consumers’ purchase decisions and consumption practices [15]. For this reason, marketing managers spend millions of dollars researching consumer attitudes toward products and brands and spend many more trying to influence these attitudes. By influencing consumers’ attitudes, marketers hope to influence consumers’ purchase behavior [16]. Attitudes are also suggested to be one of the main determinants explaining food consumption behavior, including seafood consumption behavior [17].

Since fish consumption has been associated with the healthy life concept and a balanced diet during the last decades, authorities have encouraged fish consumption [2,3,8,18,19,20,21,22,23,24,25,26,27]. The World Health Organization recommends eating at least two servings of fish per week [17,21,28].

From 1961 to 2017, global fish consumption increased by 3.1% at a rate nearly twice that of the annual world population growth (1.6%) and faster than all other animal protein foods (meat, dairy, milk, etc.), which increased at a rate of 2.1% per year. Per capita, fish consumption increased by about 1.5% per year from 9.0 kg (live weight equivalent) in 1961 to 20.5 kg in 2018. In 2017, fish consumption accounted for 17% of the global population’s animal protein consumption and 7% of the total protein consumption [19,29,30]. The increase in the world population with higher living standards, efforts of consumers in healthy nutritional consumption, improvements in seafood processing technologies, and promotion campaigns, which changed consumption habits, have led to an increase in fish consumption in recent years [31,32,33,34,35]. Thus, the increased fish consumption has directed marketers and researchers to focus more on consumers’ fish consumption behavior.

Consumer attitudes significantly impact fish consumption and buying decisions, so there is significant interest in learning more about the dynamics of choice behavior for seafood [20,32]. Seafood consumption is influenced by many interrelating factors, including attitudes, personal factors, and the cultural and social environment [36]. The attitudes that may affect individuals’ fish purchase and consumption behavior have been studied previously. In this context, the attributes and patterns for fish consumption vary across cultures, countries, and individuals. The most commonly studied attributes are the taste, texture, nutritional value, price, familiarity, health, convenience, availability, season, geographical origin, freshness, and obtainment method [19,25,30,34,36,37,38,39,40]. In addition to those, consumers’ sociodemographic and economic characteristics are also accepted as impacting fish consumption behavior [25,34,39,41].

Attitudes act as a mediator in consumers’ food purchasing behavior and affect the intention and consumption of a food product [42,43]. Thus, it is important to understand the relationship between attitudes and choice behavior. The demand for fish has increased in the last few decades due to the high nutritional value, increase in health problems based on nutrition in society, easier access to fish (sales to supermarkets), increase in the consumption behavior outside the home, promotional campaigns, and innovations in the form of fish consumption (such as fish fingers) [23,25,31]. In this context, understanding consumer variable demand structures in the face of increasing demand is very important for all parties involved (fish sector, policy makers, relevant non-governmental organizations, and consumers). The main focus of this research was to identify and explain the factors that affect consumers’ choice behavior towards fish within the framework of attitudes and sociodemographic characteristics. For this purpose, the fish consumption pattern and attitudes and socio-demographic characteristics that determine and affect the fish consumption and purchasing preference behaviors in Turkey were analyzed using the regression model and descriptive statistics. In the model, attitudes and sociodemographic features were utilized as a proxy for an overall assessment of fish and they referred to the degree to which consumers positively or negatively evaluate. The results provide information to researchers, policy makers, NGOs, and fish industry stakeholders by understanding and explaining the fish choice behavior [19,44,45]. Thus, they can create new effective strategies and marketing plans to create a sustainable value chain for fish consumption.

## 2. Material and Methods

### 2.1. Data Description

The quantitative data required to present the consumers’ fish purchase and consumption patterns and explain the relationship between attitudes and fish choice behavior were gathered by a cross-sectional survey. This survey was conducted in the major cities (Adana, Ankara, Gaziantep, İstanbul, İzmir, Trabzon, and Van) of the seven geographical regions of Turkey during August–October 2021 by a commercial marketing agency (Ayna Market Research Co.). These cities were chosen due to their geographical dispersion and population sizes, making them a good representation of the entire country. The sample size allocated to each city was determined according to their respective populations. The questionnaire was applied to 450 consumers who were responsible for their households’ food purchases. After the preliminary examination, 421 were chosen for evaluation and used in the analysis. The final sample size was extracted from an infinite population with a confidence level of 95.5% (*p* = 0.05). The sociodemographic characteristics of the sample are given in Table 1.

### 2.2. Survey Design

The questionnaire was divided into two parts. The first part was designed in four sub-sections to gather information related to the consumption patterns, attitudes toward fish choice behavior, and sociodemographic characteristics of the participants, which later formed the independent variables of the analysis. The participants were asked to score the importance of each item on a four- or five-point Likert scale to express how strongly they agreed or disagreed with each statement in this part.

The second part consisted of the question ‘How often do you consume fish?’, which constituted the model’s dependent variable. The dependent variable, which is expressed by the consumption frequency, was formed to take the value of ‘1’ if fish consumption was every day or 2–3 times a week, ‘2’ if it was once a week or 2–3 times a month, and ‘3’ if it was once a month or less than once a month (Table 2).

### 2.3. Analytical Framework and the Model

The data gathered through the survey application were analyzed using descriptive statistics and a regression model. The impacts of attitudes on consumer choice behavior have been frequently analyzed by regression models in previous studies [42,43,46,47].

In the model, to determine the factors affecting the frequency of individuals’ fish consumption, the answers given to the question ‘How often do you consume fish?’ in the survey were determined as the dependent variable. In line with the answers given by the individuals, the dependent variable was classified into three categories: ‘often’ if it was every day or 2–3 times a week, ‘sometimes’ if it was once a week or 2–3 times a month, and ‘rarely’ if it was once a month or less than once a month (see Table 2). In this context, since the dependent variable consists of more than two values and has a natural ordering, it is appropriate to apply the ordered probit model to the variables [48].

Generally, the ordered probit model can be specified from the unobserved latent variable $\left(Y_{i}^{*}\right)$:

(1) $$Y_{i}^{*}=x_{i}^{\prime }\beta +\varepsilon_{i}$$

where i (i=1,2,…,N) denotes consumers, $x_{i}$ is the vector of independent explanatory variables, β is the vector of unknown slope parameters, and $\varepsilon_{i}$ is an unobserved individual error term with a standard logistic distribution. The answers given to the question ‘How often do you consume fish?’, which is the dependent variable expressed by the consumption frequency in the model, were coded as shown in Table 2 and used in this form in the analysis. So, lower levels of $Y_{i}^{*}$ indicated that the individual has a higher consumption frequency. In comparison, higher levels of $Y_{i}^{*}$ indicated that an individual has a lower consumption frequency. In short, in the ordered probit model, the dependent variable (i.e., the consumption frequency of individuals) was classified according to the values that the unobserved latent variable $\left(Y_{i}^{*}\right)$ takes within certain threshold value ranges $\tau_{k}$:

(2) $$Y_{i}=\left\{\begin{array}{c} 1,\\ 2,\\ 3, \end{array}\right.\begin{array}{c} \prime often\prime \\ \prime sometimes\prime \\ \prime rarely\prime \end{array}\begin{array}{c} if\\ if\\ if \end{array}\begin{array}{c} \tau_{0}<Y_{i}^{*}\leq \tau_{1}\\ \tau_{1}<Y_{i}^{*}\leq \tau_{2}\\ \tau_{2}<Y_{i}^{*}\leq \tau_{3} \end{array}$$

where $\tau_{k}\left(k=1,2,3\right)$ indicates the threshold level according to the consumption frequency of each individual. In addition, the probability of choosing a particular consumption frequency of each individual is as follows:

(3) $$P(Y_{i}=j|x_{i},\beta )=P\left(\tau_{j-1}<Y_{i}^{*}\leq \tau_{j}\right)\;\;\;\;\;\;\;\;=P\left(\tau_{j-1}<x_{i}^{\prime }\beta +\varepsilon_{i}\leq \tau_{j}\right)\;\;\;\;\;\;\;\;\;\;=P\left(\tau_{j-1}-x_{i}^{\prime }\beta <\varepsilon_{i}\leq \tau_{j}-x_{i}^{\prime }\beta \right)\;\;\;\;\;\;\;\;\;\;=F\left(\tau_{j}-x_{i}^{\prime }\beta \right)-F\left(\tau_{j-1}-x_{i}^{\prime }\beta \right)$$

where F is the cumulative distribution function of $\varepsilon_{i}$ [49]. To calculate the explanatory variables’ effects on each individual’s preference probability, the derivative of the cumulative distribution specified in Equation (3) was taken according to the explanatory variables. The model estimation was carried out using the maximum likelihood method, and the logarithmic likelihood function is expressed by Equation (4) [50,51]:

(4) $$\ln L(\beta ,\tau |y,x)=\overset{J}{\underset{j=1}{\sum }}\underset{Y_{i}=j}{\sum }\ln[F\left(\tau_{j}-x_{i}^{\prime }\beta \right)-F\left(\tau_{j-1}-x_{i}^{\prime }\beta \right)]$$

A principal component analysis (PCA) with a varimax rotation was applied to dimensionality reduction in order to reduce the complexity of the model. Thus, it was possible to determine the basic dimensions of consumer participation in fish selection behavior [31,52,53,54]. Variables with factor loadings of ≥0.4 were removed from the factor set [55]. With the help of this dimensional reduction with PCA, 23 attitudes in the survey were reduced to 15.

The level of significance was set at *p* < 0.05. To perform the data analysis, ordered probit regression was conducted using StataCorp. 2013. Stata Statistical Software (STATA) v13 (College Station, TX, USA: StataCorp LP.) and PCA, and descriptive statistics (mean values) were reached using Social Sciences (SPSS) v21.0 software (Armonk, NY, USA: IBM Corp.) [56].

## 3. Results

### 3.1. Descriptive Statistics Results

According to Table 2, 11.6% of the individuals in our sample consumed fish daily or 2–3 times a week, 56.8% once a week or 2–3 times a month, and 31.6% once a month or less than once a month.

**Figure 1:**
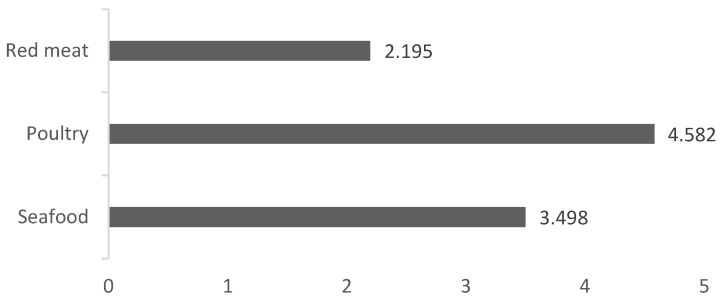
Monthly household meat consumption distribution (kg).

In this study, the general meat consumption distribution of the individuals was determined, and it was concluded that the average fish consumption was less than the consumption of red meat and more than the consumption of poultry (3.5 kg per month) (Figure 1).

**Figure 2:**
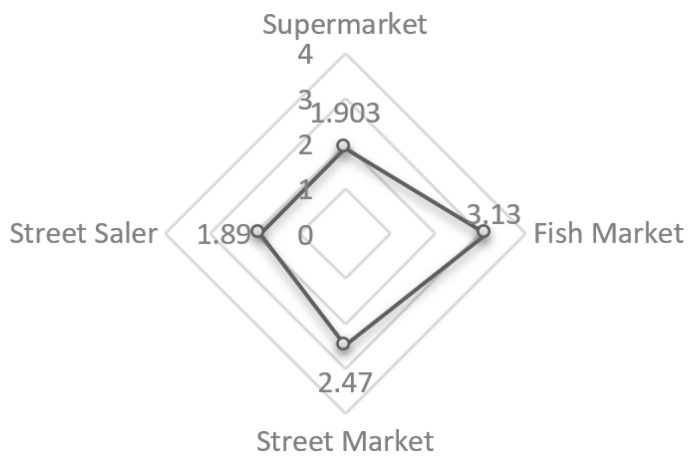
Fish purchase location preferences.

In this research, consumers’ purchasing place preferences for where to buy fish were also determined (Figure 2). Accordingly, it is understood that consumers in Turkey mostly buy fish from fish markets. Street markets, supermarkets, and street vendors came after fish markets in the purchasing place preferences.

**Figure 3:**
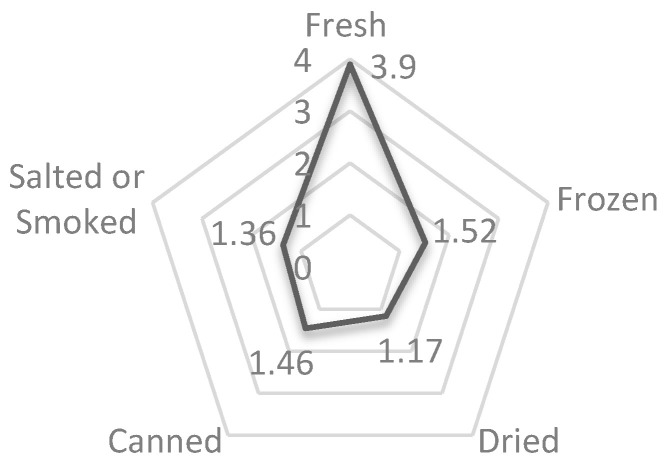
Fish consumption preferences.

Figure 3 shows consumers’ fish consumption preferences. Accordingly, consumers mostly preferred to consume fresh fish instead of processed options (canned, frozen, dried, salted, or smoked).

The results obtained within the scope of this research show that more than half of the consumers consume fish once a week or 2–3 times a month. Consumers prefer fish markets for their fish purchases, and their consumption preferences are mostly for fresh fish.

The description and mean values of the socio-economic characteristics of the sampled individuals are given in Table 3. According to Table 3, the mean value of the gender variable is 0.524, indicating that 52% of the individuals in this study were female. The mean value of the education variable is 2.857. Accordingly, most individuals in the sample were primary and high school graduates. The average value of the income variable is 3.080, which indicates that the monthly income of the individuals in the sample generally varies between 3000 and 4500 TL. The average household size was found to be three to four individuals.

Table 4 presents the definitions, mean, and standard errors of this study’s explanatory variables (attitudes). Accordingly, the average values of the variables of taste, price, accessibility, traditional consumption, variety, seller trust, production method, and physical appearance were found to be above a score of four. According to this, the individuals in the sample generally showed a traditional approach to fish consumption by finding the fish meat delicious, the price of the fish high, and the fish easy to access or buy. Trust in the seller, production method, and the physical appearance of the fish they buy or consume were also important for the individuals participating in the sampling.

### 3.2. Regression Model Results

Table 5 shows the results of the ordered probit analysis regarding the relationships between the attitudes toward fish choice behavior, sociodemographic characteristics of the participants, and frequency of fish consumption. Table 5 also demonstrates the coefficient estimates of the explanatory variables in the ordered probit model. In this study, the dependent variable, which expresses the fish consumption frequency of individuals, takes the values of 1, 2, and 3, where 1 represents the situations where the frequency of fish consumption is the least, 2 represents the medium level, and 3 represents the situations where it is the most.

The answers given to the questions regarding the attitudes toward fish consumption of the individuals are presented in Table 4 and Table 5, where strongly disagree = 1, disagree = 2, undecided = 3, agree = 4, and completely agree = 5. When the regression results were examined, statistically significant relationships were found between the attitudes, taste, price, convenience, seller trust, wild fish, physical appearance, and fish consumption frequency. In addition, a significant relationship between the education variable, one of the sociodemographic variables, and the frequency of fish consumption was found. Other attitudes about fish consumption and sociodemographic variables were not found to be statistically significant with the frequency of fish consumption.

## 4. Discussion

Within the scope of this research, all attitudes that may be effective in fish consumption were evaluated, and those related to consumers’ purchase or consumption were determined with the help of the established probit model. Accordingly, taste, convenience, seller confidence, wild fish, and physical appearance attitudes were found to be positively related to fish purchase and consumption while the price was negatively related. In addition, the effect of sociodemographic characteristics on fish consumption or purchase was also included in this research model. In this context, a positive relationship between education level and fish consumption behavior was revealed.

According to the results of the descriptive statistics, monthly fish consumption was determined to be higher than red meat and lower than poultry consumption. Middle-income families in Turkey mostly consume poultry for their meat requirements. The relatively low price of poultry is the main reason for this preference, especially in times of high inflation such as now. In addition, fish are mostly preferred by consumers with higher education levels [25,31,57,58]. Due to its high price, red meat consumption is lower than the consumption of fish and poultry.

The descriptive statistics results also show that consumers mostly prefer fish markets for their fish purchases. Among the factors that cause the preference for purchasing in fish markets is the abundance of fish varieties, high probability of finding fresh fish, and relatively lower prices. The fact that fresh fish is the first preference supports the finding that individuals prefer fish markets, which are one of the first links in the supply chain and provide access to fresh fish at lower prices. This is also supported by the correlation between the purchase frequency and the price factor in the regression results.

The majority of consumers preferred fresh fish instead of processed options. Considering the consumption habits of Turkish consumers, dried, salted, and smoked fish are much less preferred than fresh, canned, and frozen fish. Fresh fish is cheaper than processed fish, and habits and cultural factors are effective in the prominence of fresh fish as a fish consumption preference. In their studies, [8] also stated that freshness is one of the most important determinants affecting the level of fish consumption.

According to the ordered probit regression results revealing the relationships between attitudes toward fish and the probability of fish consumption (see Table 5), the attitude with the highest significance with the frequency of fish consumption was physical appearance. The greater the importance given to the physical appearance of the fish, the more likely an individual is to consume or purchase fish more often. In their study, [39] established a relationship between the physical appearance of fish and consumer confidence in fresh seafood. Moreover, [36] reported that one of the attitudes that has the highest impact on the measured perceived quality is the physical appearance of the fish, along with taste and texture. The study of [59] also stated that the indicators of freshness, physical appearance, and color were found to be significant in terms of consumer attitudes.

There is strong evidence that taste is a good predictor of consumers’ food choices as a sensory attribute. Consumers often mention taste as a major factor in their food preference decisions [60,61,62,63]. The research of [64] was one of the first to reveal the importance of taste attitudes by concluding that husbands, rather than wives, priorities the taste factor in their food preferences.

In many studies on fish consumption preferences, the importance of the taste variable for consumers has also been revealed. In their studies, [3,18,20,27,31,36,37,39,65,66,67,68,69] found that taste is related to fish or other types of seafood purchase and consumption behaviors. The probit model results of the current study also show that the more delicious consumers find the fish, the more they increase their consumption.

In many studies, price was determined as the leading factor among the barriers to fish consumption, together with the trust of the seller, presence of fishbone, availability, and smell [18,20,27,31,36,68,69,70,71]. Fish is perceived to be expensive in most countries, and if consumers indicate that the price level is important, their intention to purchase fish may decrease [37,57]. The low price of fresh fish in the market is probably an advantage for the product to be selected or substituted for other foods, especially in developing countries where high inflation rates are effective such as Turkey [35,36]. Additionally, in our study, price was a significant factor in fish choice behavior. In their study, [72] reached similar results: the probability of consuming fish decreased by 3.4% if the consumers perceived the price as being high.

As previously mentioned, trust is another barrier to consuming fish or seafood products. Lack of trust in choosing and preparing seafood is one of the biggest barriers to seafood consumption [20,34]. Consumer confidence in products moderates the relationship between attitudes and behavioral intentions [73]. In Turkey, consumers prefer fish markets instead of organized retail stores such as supermarkets as the place to buy fish, which reveals the trust factor. In fish markets, on the other hand, there is no intermediary assurance mechanism in determining the qualities of fish, such as freshness and being natural. The seller’s declaration is the only element of trust. Therefore, seller reliability is very important. The research results also show a negative relationship between consumers giving importance to the seller’s reliability when purchasing fish and fish consumption. As the trust in the seller decreases, the probability of purchasing decreases.

In fish choice behavior, convenience is accepted as another important factor and can determine whether an individual consumes fish or not [39]. The convenience attitude was detected as being significant in previous studies and accepted as a controlling factor determining the seafood choice [2,34,36,37,39,65,74]. The authors of [67] found that convenience is the second most important attitude for fish consumption after shelf life. The authors of [20] indicated that convenience is one of the leading drivers of seafood consumption in Australia. Due to the role of the convenience attitude, there is a demand for fish consumption in a frozen or canned form, which is ready to be cooked or eaten [34]. In our study, the convenience attitude is significantly related to fish consumption. As the consumers perceive that the preparation or consumption fish to be convenient, the probability of consuming or purchasing fish will increase.

In the seafood sector, fish are sourced from aquaculture farms or caught in the wild [2]. In many fish markets, wild fish have a more favorable image regarding taste, health, and nutritional value than aquaculture fish [2,26,40,75]. The quality perception of wild fish is also higher than aquaculture fish. Consumers have more positive attitudes toward wild fish and are willing to pay a premium for wild fish [2,26]. The probit model of this study also established a relation between wild fish preferences and an increase in consumption. According to the model results, the fact that the fish is wild rather than from aquaculture increases the probability of consumption.

The effects of sociodemographic variables on the frequency of consumption were also evaluated within the scope of this study. The results show that among the variables of gender, age, education, income, and household size, education is the only variable associated with the consumption frequency. The positive relationship between education and consumption frequency shows that the higher the education level of the consumers, the higher the probability of an increase in the fish consumption frequency. This is in line with previous studies of [25,28,57,69,76,77], which all found a positive association between education and fish consumption behavior.

## 5. Conclusions

It can be concluded from the results of this study that greater importance should be given to the modernization of fish markets and more efficient supply chains should be established to deliver fresh fish products to consumers in Turkey. Promotional activities should be carried out for canned and frozen products, which have important industrial infrastructure opportunities in Turkey. Thus, convenience, an important attitude regarding fish, can also be achieved. Fish should be made to be a more accessible and consumable product by applying policies to reduce consumer prices. In Turkey, fish is usually cooked and served without using diverse receipts and culinary arts. We can state that applications that enhance the flavor of fish (such as new cooking methods, sauces, or recipes) will positively affect fish consumption. Diversity and value should be added to fish presentation by researching fish dishes and serving methods in countries with high fish consumption habits. Since consumers give importance to the physical appearance of fish, and this attitude is related to consumption, the appearance characteristics of fresh fish should be highlighted in the market.

One of the main determinants of the pricing of fresh fish in Turkey is whether the fish is wild or from aquaculture. Consumers generally prefer wild fish to aquaculture fish, but wild fish costs about 20% more. There is no standard-setting application that enables them to distinguish whether the fish is wild or farmed, and they generally have to rely on the seller’s statement. Seafood fraud has recently increased by 60%, and the seafood industry has been hit particularly hard. Salmon, tuna, and halibut are just a few varieties that are commonly mislabeled in restaurants and grocery stores. A new blockchain network aims to eliminate fraud by tracking seafood from the point of capture to the kitchen. With the knowledge of where and how the fish are produced using QR code applications, the consumer can better know what they are buying. With the increase in these applications, the complexity of this matter may be solved to a great extent. However, the absence of QR codes and blockchain applications creates doubt in consumers, and their trust in the seller becomes very important. For this reason, consumers generally buy fish from people or institutions they know. Features such as the freshness or wildness of fish should be positioned according to market standards and consumer perceptions.

With the help of these results, efficient marketing strategies and promotional practices can be created, and inefficient ones can be avoided. Thus, the research findings have important managerial and policy implications that concern various stakeholders.

### Limitations

Some limitations of this study should be acknowledged. First, this study only focused on individuals’ fish purchasing behavior for their home consumption. However, it is known that there are options for away-from-home fish consumption such as fish restaurants, fast food, and cafes. Research can also be applied to the away-from-home consumption behaviors of consumers regarding fish. Second, continuing pandemic conditions during the research process forced this research to be conducted with a limited number of samples to prevent an impact on public health. Last, to avoid confusing results, only fish were focused on, neglecting other aquatic products. Similar studies can also be carried out for other types of seafood.

## Figures and Tables

**Table 1:** Sociodemographic characteristics of the sample.

**Gender**	% *	f	**Age**	% *	f	**Education**	% *	f
Female	52.5	221	≤34	42.1	177	Uneducated	5.0	21
Male	47.5	200	35–49	35.4	148	Primary	32.8	138
			50–69	21.4	90	High School	33.7	142
			≥70	1.4	6	University	28.5	120
**Income**	% *	f	**H. Size**	**%** *	f			
No Income	9.7	41	1	8.3	35			
<3000 TL	21.4	90	2	12.4	52			
3001–4500 TL	36.8	155	3	25.9	110			
4501–6000 TL	20.0	84	4	28.7	120			
6001–7500 TL	7.4	31	≥5	24.7	104			
>7501 TL	4.7	20						

* Respondent percentages for each category. H. size: Household size. TL: Turkish Lira. f: frequency of the values

**Table 2:** Categorization of the fish consumption frequencies.

Dependent Variable	f	% *
$Y_{1}$ = often	every day or 2–3 times a week	11.6
$Y_{2}$ = sometimes	once a week or 2–3 times a month	56.8
$Y_{3}$ = rarely	once a month or less	31.6

Description of each dependent variable. Answers for dependent variables were categorized into three levels: $Y_{1}$, $Y_{2}$, and $Y_{3}$. f: frequency of fish consumption. * Respondent percentages for each category.

**Table 3:** Categorization and mean values of the socio-demographic variables.

		Mean	SE
Gender	female: 1; male: 0	0.524	0.024
Age	<34: 1; 35–49: 2; 50–69: 3; >70: 4	1.817	0.039
Education	Uneducated: 1; primary school: 2; high school: 3; university: 4	2.857	0.043
Income	no income: 1; <3000 TL: 2; 3001–4500 TL: 3; 4501–6000 TL: 4; 6001–7500 TL: 5; >7501 TL: 6	3.080	0.060
H. size	single household: 1; *n* = 2: 2; *n* = 3: 3; *n* = 4: 4; *n* ≥ 5: 5	3.491	0.059

Categorization of sociodemographic variables (gender, age, education, income, and household size). SE = standard error. Values are the mean.

**Table 4:** Descriptive statistics on fish choices.

V.	Definitions	Mean	SE
Taste	I find fish delicious	4.323	0.034
Price	I find fish prices high	4.095	0.045
Accessibility	Accessibility is important in the fish supply	4.118	0.039
Health	Fish consumption is healthy	4.35	0.031
Traditionality	I perceive fish as a traditional food	4.066	0.042
Convenience	Convenience is important for fish choice	3.681	0.052
Fishing method	Fishing method is important for fish choice	3.888	0.048
Species	Species of fish is important for fish choice	4.040	0.036
Unnatural sub.	Concerned about use of unnatural sub.	3.672	0.049
Location	Location where fish is caught is important	3.94	0.041
Seller trust	trust the seller is important	4.090	0.037
Wild fish	I prefer wild fish	4.010	0.041
Freshness concern	Concerned about the freshness of the fish	3.980	0.041
Phy. appearance	I pay attention to the phy. app. of the fish	3.757	0.046

V.: Variables. SE = standard error. 5-point Likert scales: Strongly disagree (1), Disagree (2), Undecided (3), Agree (4), Strongly agree (5). Values are the mean.

**Table 5:** Ordered probit model coefficient estimates.

V.	Coeff.	SE	p
Taste	0.203	0.084	0.016 **
Price	−0.121	0.059	0.043 **
Accessibility	0.106	0.071	0.134
Health	0.069	0.087	0.425
Traditionality	−0.048	0.064	0.454
Convenience	0.109	0.051	0.035 **
Fishing method	0.032	0.061	0.594
Species	−0.117	0.073	0.111
Use of unnatural sub.	0.0431	0.057	0.434
Location	−0.043	0.068	0.526
Seller trust	0.153	0.076	0.044 **
Wild fish	−0.113	0.063	0.074 *
Freshness concern	−0.095	0.068	0.164
Physical appearance	0.182	0.058	0.002 ***
Gender	0.081	0.1050	0.439
Age	0.027	0.073	0.703
Education	0.190	0.069	0.006 ***
Income	0.047	0.0.045	0.306
Household size	−0.058	0.043	0.226
Cut1	1.556	0.667	
Cut2	3.446	0.676	

V.: independent variables of the model. Coeff: coefficients. p: prob value. SE = standard error. Level of statistical significance: * p < 0.01, ** p < 0.05, *** p < 0.010%.

## Data Availability

Data is contained within the article.
